# Recurrent Caterpillar-like Arachnoid Cysts Following Initial Resection: A Unique Presentation of a Disorder Where the Limits of Modern Medicine Are Reached

**DOI:** 10.7759/cureus.3946

**Published:** 2019-01-23

**Authors:** Sean W Kaloostian, Tara K Vartanian, Christ Ordookhanian, Talia Vartanian, Paul E Kaloostian

**Affiliations:** 1 Neurological Surgery, Haider Spine Center, Riverside, USA; 2 Internal Medicine, White Memorial Medical Center, Los Angeles, USA; 3 Biochemistry, University of California, Riverside, USA; 4 Physical Medicine and Rehabilitation, University of Southern California, Pomona, USA

**Keywords:** arachnoid cyst, caterpillar-like, craniospinal, neuraxis, recurrence, limitations, educational

## Abstract

Of the many emergent neurological cases presenting to the emergency department (ED) and operating room (OR) for resection, arachnoid cysts are amongst one of the rarer pathologies. The proper resection of arachnoid cysts has substantially decreased the risk of recurrence. Current medicinal and surgical approaches have been refined over the years and prove to be successful for many patients. Where current practices begin to fail is during the treatment of complex and rare cases, such as the one presented in this manuscript. The commonly accepted surgical practices that were utilized to aid in the management of our patient, who initially presented with a simple arachnoid cyst, unexpectedly resulted in the further development of additional arachnoid cysts, a very rare occurrence, and a complication that should be discussed amongst all specialists in the hope of identifying more focused, novel, and less-invasive approaches to cyst removal and recurrence prevention.

## Introduction

Arachnoid cysts are congenital disorders visually characterized as benign lesions physiologically represented by herniations of the arachnoid membrane through pre-existing and/or acquired dural defects [1-2]. In this report, we present an extremely rare case of an individual with contiguous pan-neuraxis craniospinal arachnoid cysts. The patient is a 49-year-old Hispanic female who initially presented with worsening right lower extremity radicular pain and urinary retention. The examination was consistent with myelopathy, and imaging revealed several contiguous cysts communicating within the subarachnoid space through loculated membranes. She successfully underwent multiple thoracic and lumbar laminectomies, resulting in the complete resolution of preoperative symptoms. After an extensive review of the literature, our patient represents a novel case of recurrent, caterpillar-like arachnoid cyst pathology of the entire spine.

## Case presentation

A 49-year-old Hispanic female presented to the clinic one month following a widening of a previous laminectomy for the resection of a lumbar (L) level four to five arachnoid cyst, with worsening right lower extremity radicular pain and urinary retention. The patient reported bilateral lower extremity pain but predominantly focused on the worsening right-sided radicular pain that resulted in a burning sensation down into the sole of her foot. The agonizing pain was complicating our patient’s ability to walk and urinate, as the need to strain in order to get proper function resulted in increased pain down in the right leg. She did, however, deny urinary incontinence, fecal incontinence, or motor weakness in the lower extremities.

**Figure 1:**
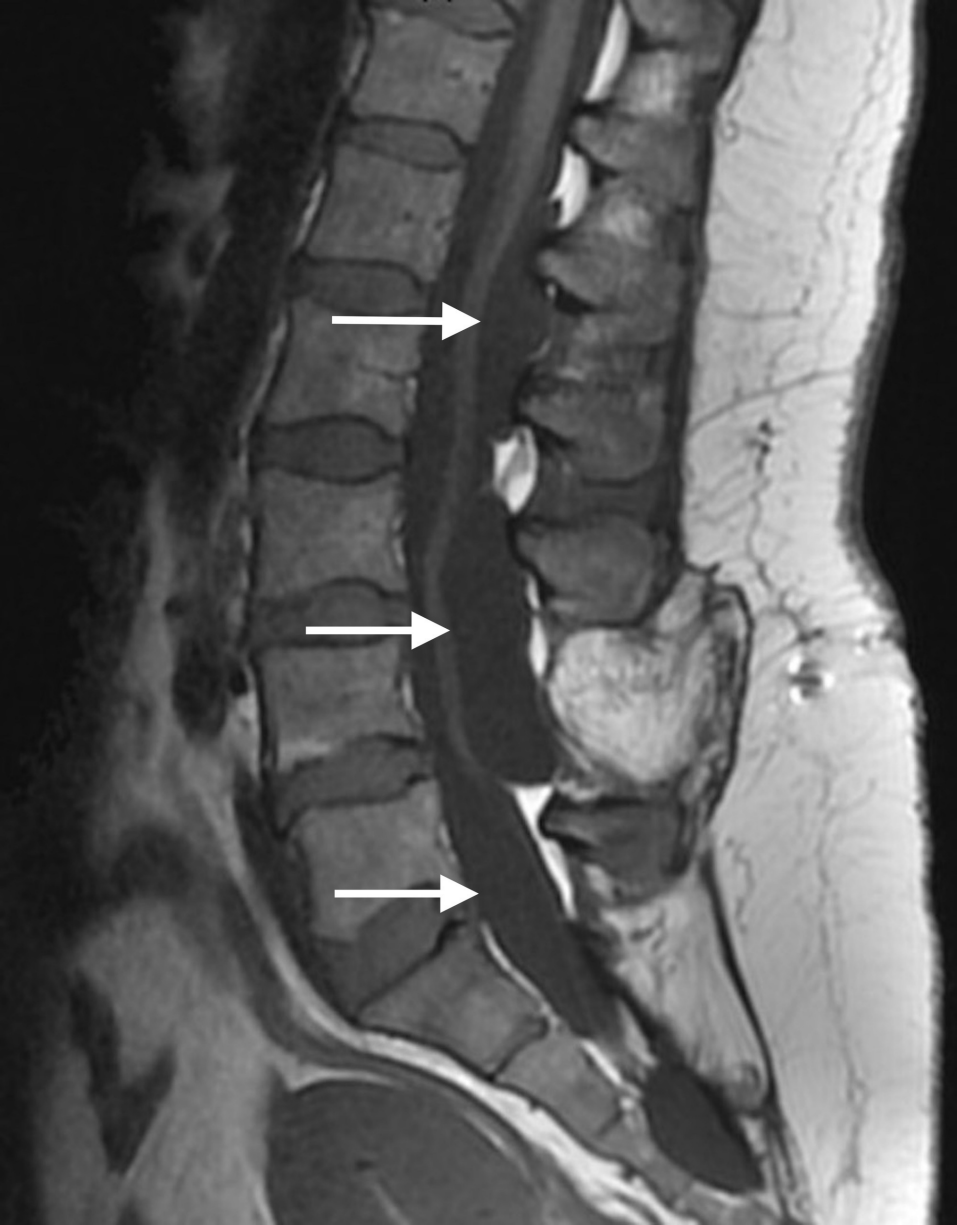
Sagittal T1 FSE MRI of the Spine FSE: fast spin echo; MRI: magnetic resonance imaging

**Figure 2:**
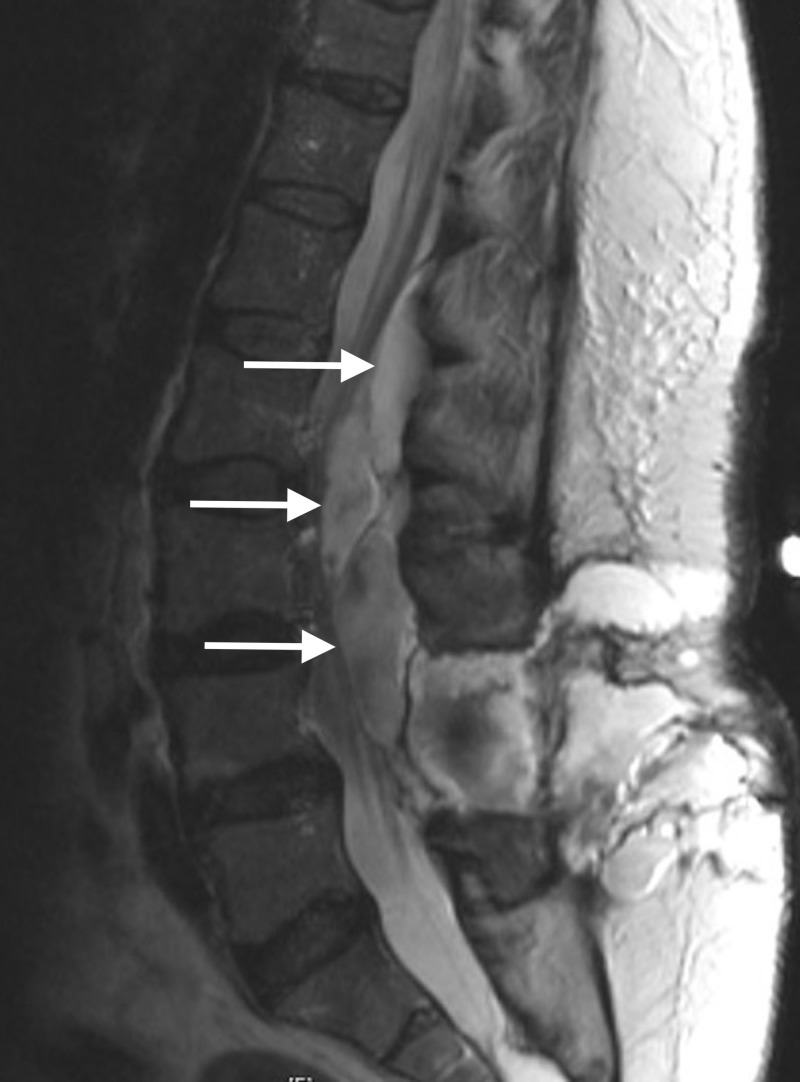
Sagittal T2 TSE MRI of the Spine TSE: turbo spin echo; MRI: magnetic resonance imaging

Magnetic resonance imaging (MRI) was obtained (Figures 1-2) and revealed caterpillar-like pan-neuraxis arachnoid cysts that initially appeared to be intradurally distributed throughout her spinal canal.

Two were visualized in the thoracic region, one extending from thoracic (T) level one to four and the other extending from thoracic level four to six. Two were localized to the lumbar region, one extending from lumbar level one to three, and the other extending from lumbar level four to five. Upon completion of a thorough neurological exam, our patient had full strength in both her upper and lower extremities without any pronator drift; a sensory exam indicated an intact system. However, on more special testing, we were able to elicit a positive straight leg raise on the right. Reflexes in the upper extremities were normal while bilateral patellar and Achilles reflexes were three-plus, suggesting myelopathy.

**Figure 3:**
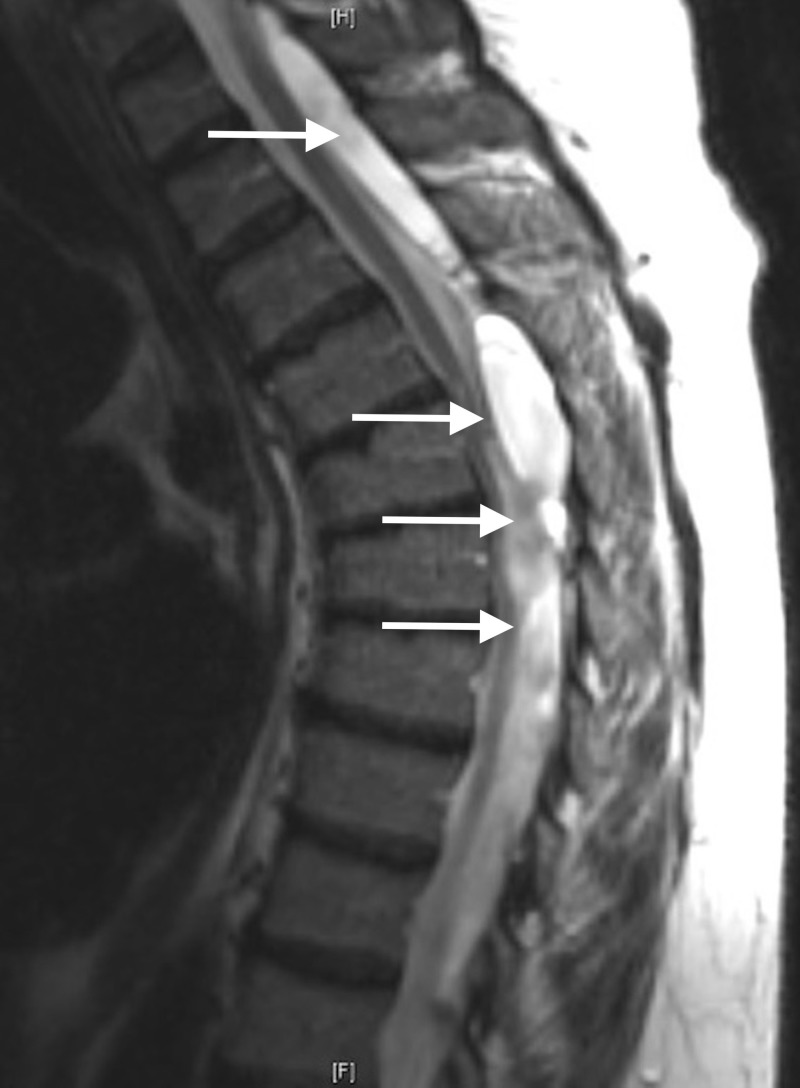
Sagittal T2 TSE MRI of the Thoracic Spine TSE: turbo spin echo; MRI: magnetic resonance imaging

MRI performed one week prior (Figure 3) showed the arachnoid cyst at thoracic level four to six. It appeared to be enlarging and exerting a mass effect on the spinal cord, causing compression with flattening of the cord at thoracic level five.

The arachnoid cyst at lumbar level one to three was noted to be enlarging and compressing the descending nerve roots more on the right side. A computed tomography (CT) myelogram was obtained the following day and revealed all caterpillar-like arachnoid cysts to be posterior and communicating separately with the subarachnoid space. Due to the patient’s difficulty with normal daily function, severe pain, and advanced myelopathy, she was brought urgently to the operating room the day after the CT myelogram was obtained for surgical intervention.

**Figure 4:**
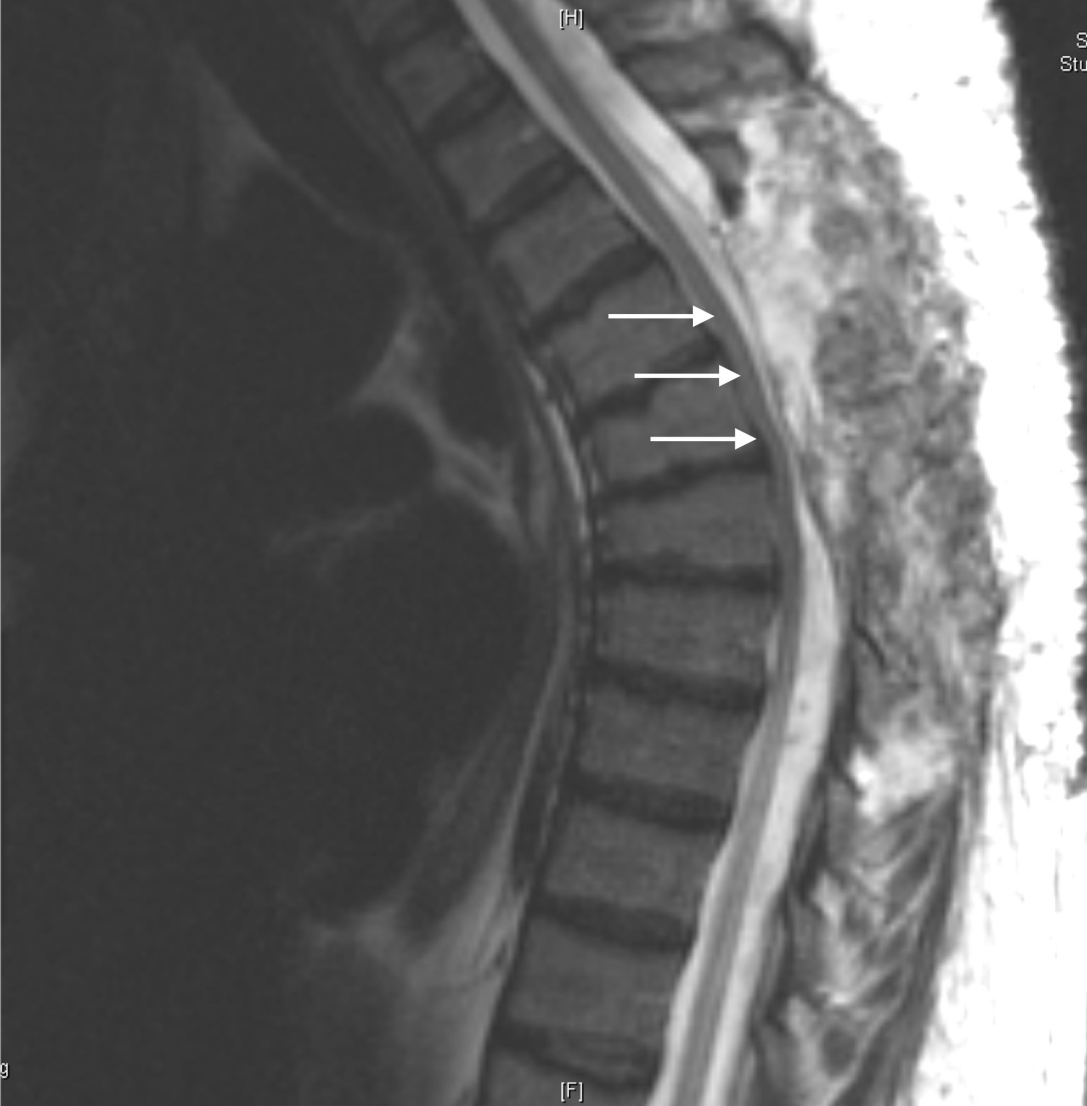
Post-Operative Sagittal MRI of Thoracic Spine

**Figure 5:**
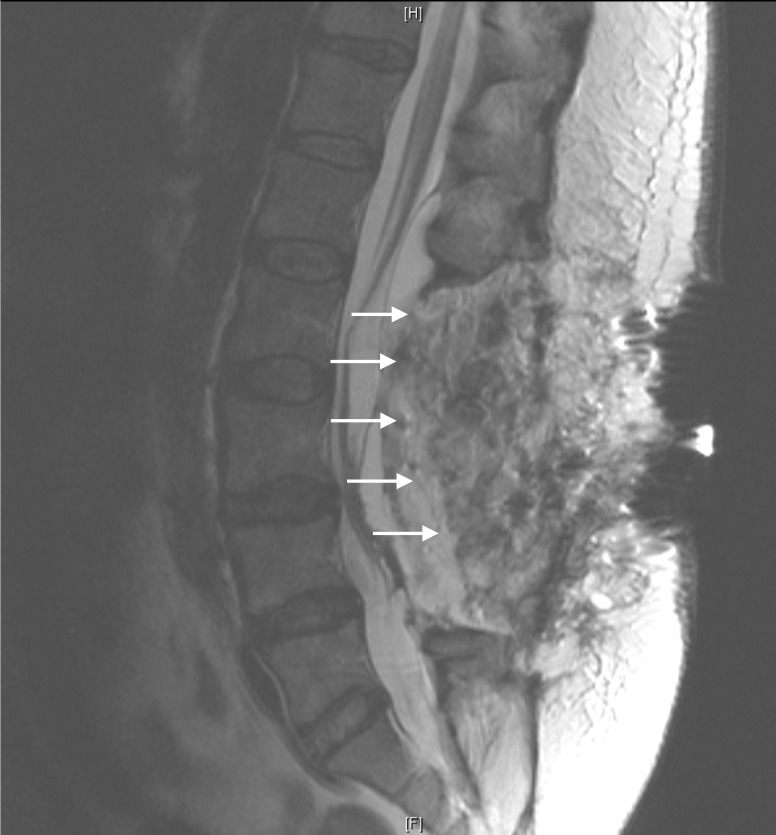
Post-Operative Sagittal MRI of Lumbar Spine

A thoracic level four to six laminectomy was planned with the excision of an intradural arachnoid cyst with dural repair. In addition, a separate incision was made for a lumbar level one to three laminectomy, with the excision of an intradural arachnoid cyst with complex dural repair at lumbar level three, the site where the patient had a prior dural defect and was found to be leaking cerebrospinal fluid (CSF). Surprisingly, at surgery, the cysts at thoracic level four to six and lumbar level one to three were noted to be extradural in nature. In both areas, we were able to work around the curved edges of the superior mass and detect the area connecting to the midline of the dura where a small area of communication within the dura was visualized. At both the thoracic level four to six and lumbar level one to three, the caterpillar-like cysts were excised epidurally and the dura was closed watertight. Postoperative MRI (Figures 4-5) revealed the unroofing of the thoracic level four to six and lumbar level one to three cysts, improvement in the caliber of the thecal sac and spinal cord, and resolution of the previously noted CSF leak.

The patient’s postoperative course was encouraging, and she was discharged on postoperative Day 5. At the one and six-month follow-up visits, the patient endorsed the improvement of preoperative symptoms, including the resolution of right lower extremity radicular pain, urinary retention, and headaches.

## Discussion

Extradural arachnoid cysts are CSF) containing cavities that are the result of the herniation of the arachnoid membrane through dural defects [3-4]. The prevalence of extradural arachnoid cysts is most likely in the thoracic cord region 65% of the time, presenting in the lumbar region 25% of the time, 6% in the sacral region, and 4% in the cervical cord region [5-6]. Adolescents tend to present with extradural cysts in the thoracic cord region while adults in their 30s and 40s typically present with lumbar or lumbosacral cysts [2,7]. Through unknown mechanisms, it is expected that approximately 50% of extradural arachnoid cysts will extend into the neural foramina, an opening between the vertebra where spinal nerve roots enter and exit [1,8-9]. Additionally, these particular cysts are twice as common in males and more likely to be symptomatic in the second decade of life [9-10]. More often than not, arachnoid cysts are asymptomatic and incidentally found through MR imaging, making it challenging to assess their true incidence, although they have been reported to represent 1%-3% of space-occupying lesions within the spinal canal [10].

Currently, there exist only a handful of adult and pediatric cases that are well documented in the literature [11-18]. However, the clinical presentation of caterpillar-like cysts throughout the pan neuraxis are extremely rare, this being the first piece of literature to describe the pathology. Our patient presented with a unique case given the recurrence of previously resected intradural cysts in the lumbar spine, in addition to the development of additional caterpillar-like cysts along the pan neuraxis causing severe neurological deficits. The pathophysiology behind the development of these lesions is still unknown and may be in part due to CSF dysregulation. To date, the best treatment, regardless of presentation, remains complete tumor resection for symptomatic cysts, which has been prevalent throughout the literature for solitary cysts [19-20]. However, our case demonstrates a rare scenario where complete surgical resection initially failed and thus resulted in more severe loculated pan neuraxis caterpillar-like cysts for the patient. Of key importance in our patient’s case was to conduct the additional screenings to determine vascularity and the CSF dynamics of each of the cysts prior to resection, which allowed for more precise surgical intervention with tactical entry points to maximize positive outcomes.

## Conclusions

Our study demonstrates the ability of such arachnoid cysts to not only form throughout the craniospinal axis but also to form multiple simultaneous loculated cysts in this region, hence the term caterpillar-like arachnoid cysts. In treating such patients, appropriate diagnostic and surgical modalities, as described above, are recommended, including the need for the imaging of the entire craniospinal axis to identify other, associated cysts that may need to be observed or treated. We believe this information will be helpful in the field of neurosurgery in terms of changing current diagnostic methods and managing patients who present with arachnoid cysts of the spine.
